# Fatal Coronary Vasculitis and Acute Myocardial Infarction Associated With Chronic Amphetamine Use: A Forensic Autopsy Case Report

**DOI:** 10.7759/cureus.112778

**Published:** 2026-07-16

**Authors:** Hatoon Hakeem, Ammar Muhyiadeen, Mohmed Hosam El-Din, Rahaf Alsayed

**Affiliations:** 1 Forensic Medicine, Ministry of Health, Madinah, SAU; 2 Toxicology, Medina Poison Control Center, Madinah, SAU; 3 Forensic Medicine, Ministry of Health, Taif, SAU

**Keywords:** acute myocardial infarction, amphetamine-induced vasculitis, forensic autopsy, forensic pathology, sudden cardiac death

## Abstract

Sudden unexpected death in young adults is a significant challenge in forensic pathology. While atherosclerotic cardiovascular disease is the most common cause of sudden cardiac death, rare chronic inflammatory conditions such as coronary vasculitis are important and often overlooked. Amphetamine use is emerging as a factor linked to severe cardiovascular complications, including arrhythmia, myocardial infarction, and vasculitis. We report the sudden death of a 27-year-old male with no medical history of cardiac illness. Postmortem autopsy revealed acute myocardial infarction with no evidence of severe coronary atherosclerosis. Histopathology of the coronary arteries demonstrated active vasculitis, with significant luminal stenosis estimated at 70-80%, accompanied by dense inflammatory infiltrates, causing disruption of the arterial wall. Blood toxicological analysis confirmed the presence of toxic amphetamine levels of 1,008 ng/mL. This case report highlights coronary vasculitis as a rare but lethal cause of sudden cardiac death. It emphasizes the need for comprehensive histopathological examination in all forensic autopsies, particularly when typical atherosclerotic findings are absent. This case report contributes to the literature on the cardiotoxic effects of amphetamine and urges forensic pathologists to maintain a high index of suspicion for drug-associated inflammatory vascular disease in the context of stimulant abuse to ensure accurate determination of the cause of death.

## Introduction

The illicit use of amphetamine-type stimulants (ATS), including methamphetamine, represents a persistent and expanding global health concern; their abuse is associated with significant morbidity and mortality [1]. The adverse cardiovascular effects of these substances are well documented and include a wide cluster of pathologies, such as dilated cardiomyopathy, myocardial infarction, aortic dissection, arrhythmias, and sudden death [2,3]. Postmortem examination of individuals with a history of amphetamine abuse frequently demonstrates cardiac abnormalities, with common histopathological findings of myocardial fiber hypertrophy, interstitial fibrosis, accelerated atherosclerosis, and myocyte necrosis [1,4]. While the link between amphetamine use and cerebrovascular accidents, including those caused by ruptured berry aneurysm, cerebral vasculitis, or necrotizing angiitis, has been described in case reports, this finding remains relatively uncommon [5]. The inflammatory effects of amphetamines on the vasculature are a recognized, yet rare, phenomenon [5]. However, a similar pathological process in the coronary arteries is not well-established in the forensic literature. Previous case reports of fatal coronary events in amphetamine users have identified pathologies such as occlusive intimal hyperplasia rather than primary vasculitis [2]. While mechanisms such as coronary artery spasm and thrombosis are often implicated in amphetamine associated myocardial infarction [6,7], vasculitis is a less frequently documented pathological finding.

The current case report documents a rare case of myocardial infarction associated with coronary vasculitis due to amphetamine use. In this case, structural failure of the vascular wall was the primary driver of the terminal event. This case report aims to provide a comprehensive forensic medicine reference. It links the clinical presentation and toxicological findings with the microscopic structural damage that characterizes this rare condition, highlighting a rare mechanism of death that may be overlooked in routine autopsies where the coronary arteries appear non-occluded.

## Case presentation

A deceased young male, approximately in the third decade of life, was brought to the Regional Forensic Medicine Center in Al-Madinah following a medico-legal request for external examination and full autopsy after sustaining sudden cardiac death. Notification of death occurred on 12/25, with forensic examination performed on 12/25 at 11:00 AM under official orders from law-enforcement authorities and the Public Prosecution.

Initial condition and external examination

Upon removal from refrigeration, the body exhibited pronounced rigor mortis, and post-mortem hypostasis was fixed, purple in color, on the back of the body. The deceased was of medium build of 80 kg and measured approximately 170 cm in height.

**Figure 1:**
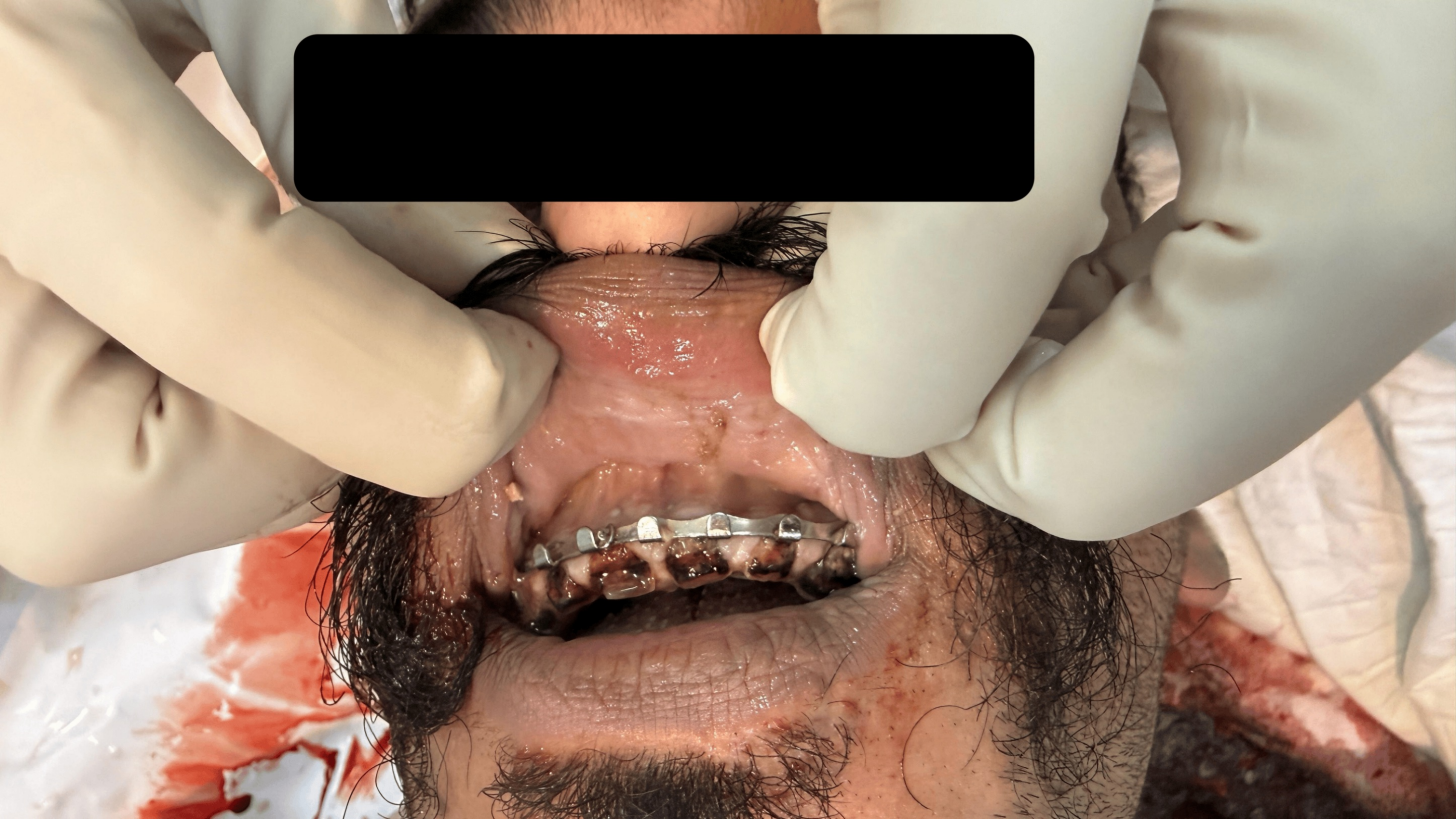
External examination of the mouth revealed severe tooth decay, known as “meth mouth."

External examination revealed severe tooth decay (Figure 1); no external injuries indicative of assault, struggle, restraint, or defensive action were identified. The body was naked with no clothes provided, and no evidence suggestive of physical violence was observed. Likewise, no patterned injuries, petechial hemorrhages, or neck trauma were present.

Internal examination

Opening of the cranial cavity revealed no fractures, intracranial bleeding, or recent traumatic lesions of the scalp, skull, or brain. Notably, the brain weighed 1,504 g, which sits at the upper physiological percentile for an adult male of his stature [8], showing marked congestion and edema, but no contusions, lacerations, or structural herniation.

Chest findings

**Figure 2:**
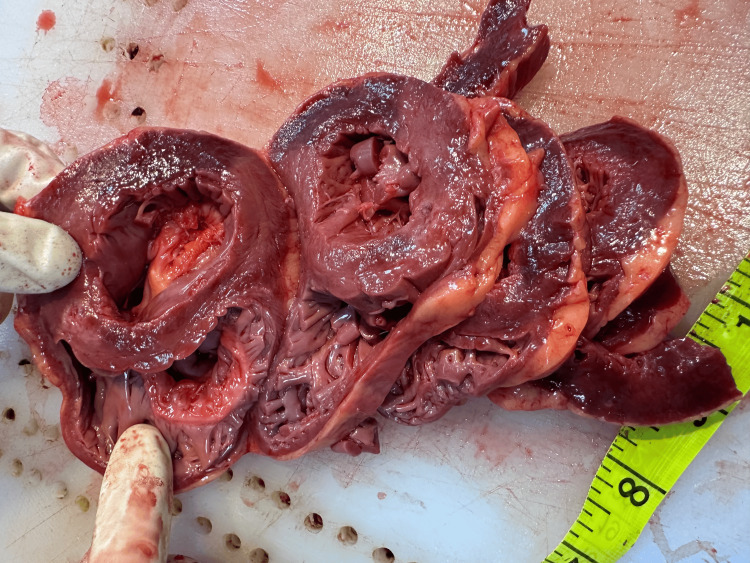
Gross examination of the myocardium demonstrated patchy dark areas of discoloration involving both ventricles and the interventricular septum, consistent with acute myocardial infarction.

**Figure 3:**
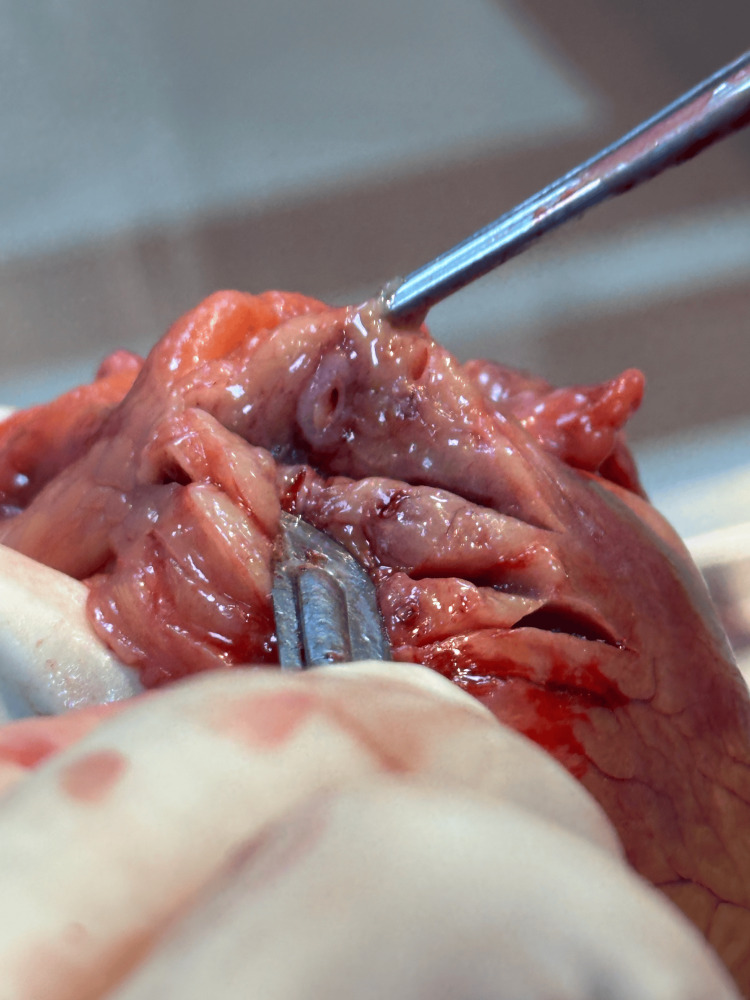
Gross examination of the left anterior descending artery showing luminal stenosis.

Significant pathology emerged upon inspection of the chest cavity; the heart weighed 370 g, which represents a mild to moderate cardiomegaly relative to the individual’s body weight and height, consistent with chronic stimulant-induced remodeling. The wall thickness of the left and right ventricles was about 1.4 cm and 0.4 cm, respectively. Serial coronary artery sectioning demonstrated diffuse multivessel coronary luminal stenosis estimated at approximately 60% involving the major coronary arteries (Figure 2). Gross examination of the myocardium revealed patchy dark discoloration involving both ventricles and the interventricular septum (Figure 3). The left and right lungs weighed 1060 g and 1100 g, respectively. These weights significantly exceed the standard forensic reference ranges for young adult males. Cut sections of the lungs showed extensive congestion. The aorta was normal, with no injuries or gross pathologies seen.

Abdominal organs, including the liver (1728 g) and the right (132 g) and left (138 g) kidneys, demonstrated congestion throughout. Additionally, no mediastinal hemorrhage was noted.

Toxicology results

Blood, urine, and gastric content in the first screening method revealed positive results for amphetamine and negative results for alcohol, opiates, benzodiazepines, barbiturates, cocaine, and cannabis. Subsequently, confirmatory steps using the gas chromatography-mass spectrometry (GC-MS) machine produced the results shown in Table 1.

**Table 1:** Quantitative amphetamine levels. Note: Concentrations verified via gas chromatography-mass spectrometry (GC-MS). Reference ranges compiled from standard forensic toxicology literature: Schulz et al. (2020). *Therapeutic and toxic ranges may overlap significantly in chronic stimulant abusers due to the development of profound physiological drug tolerance.

Sample	Amphetamine level	*Reference range and forensic interpretation
Blood (peripheral)	1,008 ng/mL	Toxic/lethal range (therapeutic: 20-150 ng/mL; toxic/lethal: > 200-1,000+ ng/mL). Indicates severe, fatal acute cardiotoxicity [9].
Urine	36,070 ng/mL	Markedly elevated. Reflects extensive renal excretion consistent with heavy, chronic usage, and recent high-dose exposure.
Stomach contents	4,506 ng/mL	Significant acute ingestion. Confirms substantial oral consumption of the substance shortly before death.

Histopathology examination

**Figure 4:**
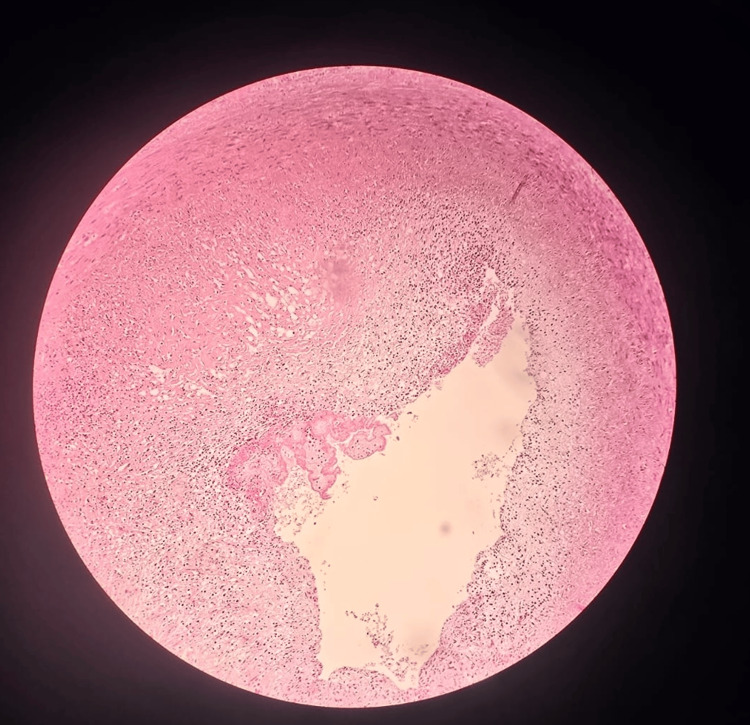
Histopathological section of the left anterior descending coronary artery showing edematous and inflamed intima with endothelial injury, fibrinous exudate, and prominent eosinophilic inflammatory infiltrates causing marked luminal narrowing, consistent with coronary vasculitis (hematoxylin and eosin stain).

Left main coronary, left anterior descending artery, and left circumflex showed edematous, inflamed intima with endothelial damage, fibrinous exudate, and eosinophilic-rich inflammatory infiltrates with associated stenosis of 70-80% (coronary vasculitis) (Figure 4). Furthermore, there was microscopic evidence of acute myocardial infarction in the right and left ventricles, and the interventricular septum dated six to 12 hours prior to death. No significant plaque formation was identified.

Both lungs show severe congestion.

The rest of the sample of brain, spleen, liver, kidneys, and adrenal glands were unremarkable. 

Final diagnosis

Based on the autopsy, toxicology, and histopathological findings, the cause of death was attributed to acute myocardial infarction secondary to amphetamine-induced coronary vasculitis, culminating in death.

## Discussion

This case report documents a rare instance of fatal coronary vasculitis associated with chronic amphetamine use. While cardiovascular pathology is frequently observed in amphetamine-related deaths, the presentation of primary coronary vasculitis remains exceptionally rare and under-reported [1,10]. Large autopsy studies of stimulant abusers more typically report histopathological changes, such as myocardial hypertrophy, perivascular and interstitial fibrosis, and premature and severe coronary atherosclerosis [10,11]. Akhgari et al. (2017), in a histopathological study of 100 methamphetamine-related deaths, noted cardiovascular pathology in 68% of cases, with the most common features being myocardial fiber hypertrophy and atherosclerosis, rarely vasculitis [1]. Similarly, a 2024 Thai forensic study of 120 medico-legal cases positive for methamphetamine reported coronary atherosclerosis in 55% and myocardial fiber hypertrophy in 35.8% of cases, but not coronary vasculitis [4]. Moreover, Phuangphung and Wongchanapai (2023), in a study of Thai postmortem cases positive for urinary methamphetamine, reported similar atherosclerotic and hypertrophic findings [10]. The finding of vasculitis in this case, therefore, represents a significant deviation from the commonly reported cardiac pathologies.

Amphetamines function as highly effective sympathomimetic compounds, promoting a significant release of catecholamines (dopamine, norepinephrine, and epinephrine) while concurrently inhibiting their reuptake [11]. The neurochemical surge results in profound cardiovascular stress, characterized by acute hypertension, tachycardia, and increased myocardial oxygen demand [2,12]. Prolonged exposure to amphetamine results in structural alterations of the cardiac muscle, as demonstrated in this case by an increased heart weight, with normal left ventricular wall thickness of 1.4 and 0.3 cm of the right ventricle [3,13]. These findings are consistent with concentric myocardial hypertrophy and extensive remodeling frequently observed in stimulant-related deaths [11,14].

A pivotal feature of this case is the histopathological confirmation of coronary vasculitis affecting the left main, left anterior descending, and circumflex coronary arteries. The presence of an edematous, inflamed intima and endothelial injury indicates an active, acute-on-chronic inflammatory surge in which fibrinous exudate and eosinophil-rich inflammatory infiltrates progressively compromise the vascular lumen [2,15]. In contrast to the focal plaque formation observed in regular atherosclerosis, the vasculitic progression typically involves circumferential wall thickening and smooth segmental tapering. In this case, it ultimately resulted in severe 70-80% stenosis affecting most major coronary segments: the left main, LAD, and LCX arteries [16]. This diffuse involvement mirrors the “marked lumen stenosis” and “disruption of the media and adventitial architectures” reported in other fatal cases of coronary artery vasculitis [15].

The terminal event in this case was an acute myocardial infarction, grossly identified by patchy dark areas and confirmed by histopathology across the ventricles and interventricular septum. In amphetamine users, acute myocardial infarction typically arises due to a multi-threat mechanism: accelerated atherosclerosis, acute coronary spasm, thrombosis, and, uniquely in this case, active vasculitis [12,16,17].

In evaluating this case, a rigorous differential diagnosis is essential to distinguish stimulant-associated vascular injury from other etiologies of coronary vasculitis. Systemic autoimmune vasculitis, such as polyarteritis nodosa or granulomatosis with polyangiitis, was excluded based on the strict anatomical confinement of the active inflammatory process to the coronary arterial tree, with no systemic multi-organ involvement or granulomatous lesions identified during complete autopsy dissection. Furthermore, infectious vasculitis was discounted due to the absence of localized microabscesses, bacterial or fungal aggregates on histopathology, and a lack of postmortem findings suggestive of infective endocarditis or systemic sepsis. While the eosinophilic-rich nature of the infiltrate could raise suspicion for hypersensitivity vasculitis, the absence of a history of newly introduced therapeutic drugs, systemic cutaneous rashes, or multi-organ allergic manifestations, coupled with the definitive presentation of chronic dental decay (“meth mouth”) and high toxicological concentrations, strongly favors a chronic stimulant-associated pathology rather than an acute drug hypersensitivity reaction.

The coronary findings in this case are remarkably distinct from the typical cardiovascular pathology reported in large-scale forensic series of stimulant-related deaths. A histopathological study of 100 methamphetamine-related deaths found that 68% of cases exhibited cardiovascular pathology yet rarely reported active vasculitis [1]. Similarly, a 2024 Thai forensic study of 120 medico-legal cases positive for methamphetamine reported coronary atherosclerosis in 55% and myocardial fiber hypertrophy in 35.8% of cases, but no evidence of coronary vasculitis [4]. A separate Thai forensic study of methamphetamine-positive postmortem cases similarly documented atherosclerosis, hypertrophy, and fibrosis and the predominant cardiac findings, with vasculitis absent from the pathological spectrum [10]. While focal lesions such as intimal hyperplasia have been documented in chronic amphetamine users [2], the acute, eosinophilic-rich pan-coronary vasculitis observed here remains an extreme rarity in the forensic literature.

The biological plausibility of such a reaction is, however, well-supported by the established relationship between amphetamines and cerebral vasculitis. Since the early 1980s, amphetamines have been recognized as a cause of necrotizing angiitis in the central nervous system, often manifesting as subarachnoid or intracerebral hemorrhage [18,19]. This cerebral vasculitis, characterized by segmental “beading” and transmural inflammation, is a known, yet rare, complication of stimulant abuse that can present with significant clinical delay or masquerade as other pathologies [20].

While functional mechanisms, such as acute coronary artery spasm and subsequent transient thrombosis, are heavily implicated in amphetamine-induced myocardial infarctions, they typically present with morphologically patent or minimally diseased vessels at autopsy [21]. In the current case, functional vasospasm cannot be entirely ruled out as a terminal precipitating factor; however, the presence of a profound, circumferential structural pathology, characterized by acute-on-chronic transmural inflammation and 70% to 80% fixed luminal stenosis involving the left main, LAD, and LCX arteries, indicates that structural vascular compromise was the primary driver of the fatal ischemic event.

The blood amphetamine level of 1,008 ng/mL is high and falls within ranges frequently associated with fatal outcomes, and this level in blood correlates with recent, high-dose consumption, although a chronic user may exhibit high tolerance [1,22]. Although a concentration exceeding 1000 ng/mL is frequently correlated with measurable pharmacological effects and acute impairment [23]. The exceptionally high concentration in urine (36,070 ng/mL) and gastric contents (4,506 ng/mL) strongly suggest a significant oral ingestion shortly before death [24]. In our case, this high concentration may have served as the trigger for the severe vascular response. The absence of substances, such as alcohol or benzodiazepines, confirms that the pathological and subsequent death were driven solely by the acute-on-chronic toxicological effects of amphetamine [1].

The systemic autopsy findings yield a clear picture of acute multi-organ failure; the massive weight of the lungs (1,060 g and 1,100 g), accompanied by congestion, indicates terminal acute left-sided heart failure and resultant pulmonary edema [11]. Cerebral edema (1,050 g) is a common secondary finding in stimulant-related deaths, often resulting from acute hypertensive crisis or hypoxic-ischemic injury during the terminal event [2]. Furthermore, the external findings of “meth-mouth” serve as a clinical marker of chronic use, often correlating with long-term physiological strain that produces the observed cardiac hypertrophy and coronary stenosis [12]. 

This case provides a postmortem demonstration that fatal amphetamine-associated coronary vasculitis, although rare, can cause fatal myocardial infarction and should be considered in sudden deaths among stimulant users.

## Conclusions

This case highlights a rare postmortem presentation within a forensic setting, demonstrating an acute myocardial infarction associated with multi-vessel coronary vasculitis in the context of chronic amphetamine abuse. While establishing a definitive, isolated causal relationship remains challenging within the inherent limitations of a single case report, the objective histopathological and toxicological findings strongly support this significant vascular association. Ultimately, this case underscores the critical necessity of thorough histopathological examination of the coronary vessels in stimulant-related fatalities. Pathologists should remain vigilant, as the underlying mechanism of ischemic infarction in these populations may involve active, drug-associated vasculitis rather than typical accelerated atherosclerosis or transient coronary vasospasm alone. 
